# A Portable Automatic Endpoint Detection System for Amplicons of Loop Mediated Isothermal Amplification on Microfluidic Compact Disk Platform

**DOI:** 10.3390/s150305376

**Published:** 2015-03-05

**Authors:** Shah Mukim Uddin, Fatimah Ibrahim, Abkar Ahmed Sayad, Aung Thiha, Koh Xiu Pei, Mas S. Mohktar, Uda Hashim, Jongman Cho, Kwai Lin Thong

**Affiliations:** 1 Department of Biomedical Engineering, Faculty of Engineering, University of Malaya, 50603 Kuala Lumpur, Malaysia; E-Mails: shahmukim@gmail.com (S.M.U.); abkar8819@gmail.com (A.A.S.); aungthiha.bme@gmail.com (A.T.); mas_dayana@um.edu.my (M.S.M.); minerva@inje.ac.kr (J.C.); 2 Centre for Innovation in Medical Engineering (CIME), Faculty of Engineering, University of Malaya, 50603 Kuala Lumpur, Malaysia; E-Mail: thongkl@um.edu.my; 3 Microbiology Unit, Institute of Biological Sciences, Faculty of Science, University of Malaya 50603 Kuala Lumpur, Malaysia; E-Mail: xp_527@yahoo.com; 4 Institute of Nano Electronic Engineering (INEE), Universiti Malaysia Perlis, 01000 Kangar, Perlis, Malaysia; E-Mail: uda@unimap.edu.my; 5 Department of Biomedical Engineering, Inje University, Gimhae 621-749, South Korea

**Keywords:** pathogen, *Salmonella*, food safety, diagnosis, LAMP, multiplexed detection, endpoint detection system, microfluidics, compact disc, lab-on-a-CD

## Abstract

In recent years, many improvements have been made in foodborne pathogen detection methods to reduce the impact of food contamination. Several rapid methods have been developed with biosensor devices to improve the way of performing pathogen detection. This paper presents an automated endpoint detection system for amplicons generated by loop mediated isothermal amplification (LAMP) on a microfluidic compact disk platform. The developed detection system utilizes a monochromatic ultraviolet (UV) emitter for excitation of fluorescent labeled LAMP amplicons and a color sensor to detect the emitted florescence from target. Then it processes the sensor output and displays the detection results on liquid crystal display (LCD). The sensitivity test has been performed with detection limit up to 2.5 × 10^−3^ ng/µL with different DNA concentrations of *Salmonella* bacteria. This system allows a rapid and automatic endpoint detection which could lead to the development of a point-of-care diagnosis device for foodborne pathogens detection in a resource-limited environment.

## 1. Introduction

Illnesses and deaths caused by foodborne microbial pathogens have received major attention worldwide. Hence, food safety is a serious public health issue. In 2013, Foodborne Diseases Active Surveillance Network (FoodNet) reported 19,056 cases of infections, 4200 hospitalizations, and 80 deaths in 10 U.S. sites (affecting approximately 15% of the U.S. population) due to foodborne diseases [1]. Failure to detect foodborne pathogens in contaminated food may not only lead to far-reaching consequences on human health but also causes a large economic burden on the food industry. Research on rapid and reliable method of foodborne pathogen detection is still on going.

Conventional methods available for the detection and identification of microbial pathogenic agents are based on selective microbiological media to isolate the viable bacterial cells present in food [2,3]. As these media depend on the ability of the organisms to multiply to become visible colonies, it requires 2–3 days to have probable results and up to 5–7 days for confirmation [3,4,5,6]. Moreover, it is labor intensive and cumbersome as the analysis of samples require several steps e.g., culture medium preparation, inoculation of plates and colony counting [2].

For sensitive detection of pathogens with automated or semi-automated instruments in near real time, several biosensors and bio-based methods have recently been developed. These include bioluminescence sensors [2], Surface Plasmon Resonance (SPR) sensors [6,7,8], electrochemical immunosensors [9], Fluorescence Resonance Energy Transfer (FRET) [5,10,11,12], piezoelectric biosensors [13] and cell based sensors [14,15,16]. In order to reduce analysis period and achieve confirmation results for detection, several methods like the Polymerase Chain Reaction (PCR) [17,18,19,20], immunoassay [21,22,23] and microarray [24] have been developed. However, these methods and techniques require complex manual steps, skilled personnel and high-end sophisticated equipment. These requirements limit the accessibility of these techniques especially in resource-poor areas. Besides, most centralized and modern laboratories are limited to large cities. Therefore, near-patient tests that utilize cheap and reliable Point of Care Test (POCT) devices have become increasingly important to provide an alternative rapid, sensitive, accurate and automated method for detection of foodborne pathogens.

Lab-on-a-Chip (LOC) device also called “Micro Total Analysis System” (µTAS) is a reliable choice as a POCT device. Miniaturized device size, low consumption of reagent and sample, precise microfluidic volume control, less manual handling errors and automated fast turn-around time for operation of biochemical assay enable the integration of all necessary processes and laboratory operations into a single chip [25,26]. These compact devices can analyze the samples at the point of need rather than in a centralized highly equipped laboratory. Various researches have been conducted on microfluidic chips for pathogens detection [27,28,29,30,31,32]. Easley *et al.* [29] reported a microfluidic genetic analysis system for detection of *Bacillus anthracis* by solid-phase extraction (SPE), PCR and microchip electrophoresis (ME) in less than 30 min. Beyor *et al.* [30] developed a laboratory-on-a-chip system for the detection of *Escherichia coli* K12 and *E. coli* O157 integrating cell pre-concentration, purification, PCR and capillary electrophoretic (CE) analysis on one single platform. Even though steps for pathogen detection start from target separation to endpoint detection are integrated on a single chip, it requires complicated tube connections for pneumatic fluid control which limits the miniaturization of full system.

Comparing all the LOC platform, lab-on-a-CD platform has received significant attention for its potential to integrate all biochemical reactions onto a single disc-pattern microfluidic device and perform multiplexed operations using a single motor. It leads toward the development of microfluidic sample-to-answer systems or µTAS *in vitro* diagnostics (IVD) [33,34]. The centrifugal, inertial and coriolis forces generated by the disk rotation can be employed for fluidic manipulation *i.e.*, pumping [35], mixing [36,37], metering [38,39], decanting [40], calibrating [40], sample splitting [40], fluid separating [40] and valving [41]. Several biochemical operations have been implemented successfully on microfluidic compact disk such as biochemical analysis and immunoassay [42], detection of protein [43], fluorescence immunoassays [44], DNA extraction [45], nutrients determination in water [46], ELISA system [47,48] and foodborne pathogen detection [49].

Among several isothermal nucleic acid amplification techniques, LAMP is one of most established methods [50,51]. LAMP provides a secure reaction temperature range of 60–65 °C as compared to other low temperature methods, for example—Nucleic Acid Sequence-Based Amplification (NASBA), Helicase-Dependent Amplification (HDA), Strand Displacement Amplification (SDA) and so on—because in all these methods there is a risk that the reaction is initialized before the completion of the reaction preparation [50]. Therefore LAMP would be very beneficial as a POCT diagnostic device with its high sensitivity and specificity. In recent years, Ajima Muangchuen *et al.* [52] were able to develop a colorimetric endpoint detection technique based on DNA amplification by LAMP for *Ehrlichia canis*, Wu *et al.* [53] have developed an integrated glass microdevice for LAMP, Lee *et al.* [54] have developed an integrated micro-reactor system that is able to detect Hepatitis B virus (HBV) DNA using LAMP reaction, Feiwu Li *et al.* [55] have also developed a visual and rapid LAMP assay for detection of the *cry2Ab* and *cry3A* genes in GM crops, these require either naked eye observation or UV-Visible Spectroscopy interfaced with a computer for obtaining confirmative results. Liang *et al.* [56] have reported a close-tube method using a wax-sealed fluorescent intercalator to detect LAMP product. Changchun Liu *et al.* [57] developed a single-chamber LAMP cassette to detect HIV-1 in oral fluid which requires a portable ESE optical detection system interfaced with a computer to get the confirmative results from generated graph. The developed system called micro-LAMP (μLAMP) by Fang *et al.* [58] required naked eye observation or compact real-time absorbance detection device for end-point detection which is expensive and complex system. Analyzing the current techniques for endpoint detection of LAMP reaction, it is necessary to develop an endpoint detection system which can provide automatic and digital confirmative results.

In this study, LAMP was performed in tubes with *Bst* DNA polymerase and a set of specially designed six primers which were incubated in 63 °C constant temperature for 60 min, followed by enzyme inactivation at 80 °C for 2 min to complete the reaction. We developed a low cost portable system equipped with an ultraviolet (UV) emitter and a color sensor for the purpose of automation and digitization for the endpoint detection technique of LAMP amplicons.

## 2. Methodology 

The method to develop the endpoint detection system for the LAMP amplicons comprise of three primary steps, *i.e.*, sample preparation, microfluidic disc fabrication and development of the detection system. Samples of LAMP amplicons with *Salmonella* bacteria were prepared following the tube-based method. Then a microfluidic CD featuring with detection chamber was fabricated. An electronic detection system was designed with suitable electronic components. The detection system was tested with LAMP amplicons containing different concentrations of *Salmonella* bacteria.

### 2.1. Sample Preparation 

The LAMP amplicons were prepared according to protocols provide by Notomi *et al.* [59] using Loopamp DNA Amplification Kit (Eiken Chemical Co., Ltd., Tokyo, Japan). The LAMP reactions were carried out in a total volume of 25 µL/test containing 12.5 µL of 2X reaction mix composition (40 mM Tris-HCl (pH8.8), 20 mM KCI, 16 mM MgSO_4_, 20 mM (NH_4_)_2_SO_4_, 0.2% Tween20, 1.6 M Betaine, 2.8 mM each dNTPs) provided in the kit, 40 pmol of each inner primer FIP and BIP, 5 pmol of each primer F3 and B3, 20 pmol of each loop primer LF and LB, 8 U of *Bst* DNA polymerase in volume of 1.0 µL, 2.0 µL of deionized water and 2.5 µL of DNA template of *Salmonella*. The same reaction mixture without DNA template (replaced by deionized water) was used as a negative control. The primers were designed based on the *fadA* gene of *Salmonella enterica* Typhimurium (GenBank accession number NC003197.1) using the LAMP Primer Explorer V4 software [60]. The primer sequences are proprietary information. DNA extraction was done by direct cell lysate boiling method and DNA concentration was measured using the NanoDrop 2000 UV-Vis Spectrophotometer. The LAMP reactions were carried out in a heat block at 65 °C for 60 min, followed by enzyme inactivation at 80 °C for 2 min to complete the reaction and then cooled off at 4 °C. An aliquot of 1 µL of 10-fold diluted SYBR Green I dye was added to 25 µL of amplicons of the LAMP assay. Immediately, the results could be visualized via the color change: a positive result is indicated by a color change from orange to yellowish green whilst in a negative result there is no change of color, *i.e.*, orange.

### 2.2. Disc Fabrication 

**Figure 1:**
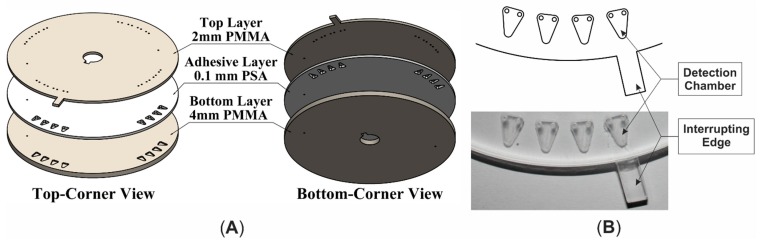
Microfluidic CD (**A**) 3D expanded view; (**B**) Top view of the featuring section.

A microfluidic compact disk (CD) was designed using a computer aided design software (*i.e.*, AutoCAD) and fabricated with layers of transparent thermoplastic of poly methyl methacrylate (PMMA) and a custom manufactured pressure sensitive adhesive (PSA). The CD composed of three layers where two layers of PMMA were bounded with a layer of PSA. Computer numerical control (CNC) machine and digitally controlled cutting plotter machine were utilized to cut the microfluidic features (chambers) in the PMMA and PSA layer, respectively. The chambers were engraved in a 4 mm thick PMMA layer (bottom layer) to load 26 µL volume of liquid. Inlets/outlets holes were cut-out in a 2 mm PMMA layer (top layer) with a shape of interrupting edge. Then the three layers were aligned and pressed-bound together using a custom made press-roller system (Figure 1).

### 2.3. Development of the Endpoint Detection System for LAMP

#### 2.3.1. Design of Detection System

**Figure 2:**
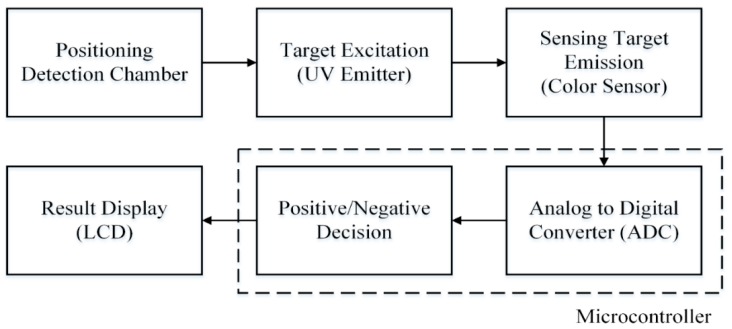
Block diagram of the endpoint detection system.

**Figure 3:**
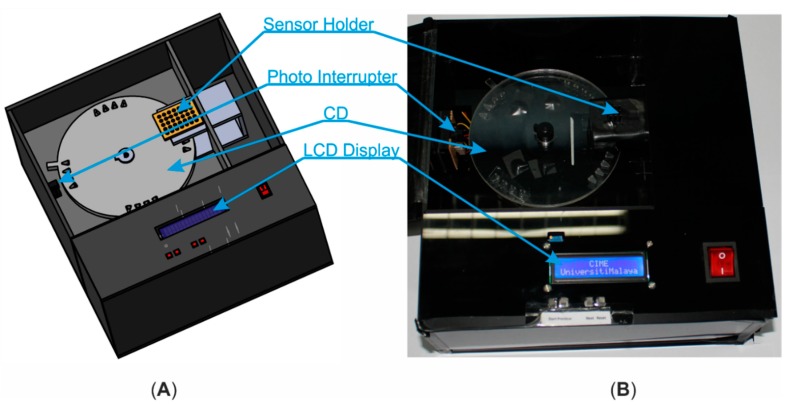
Endpoint detection system (**A**) Schematic illustration in 3D; (**B**) Photograph of the operating model.

**Figure 4:**
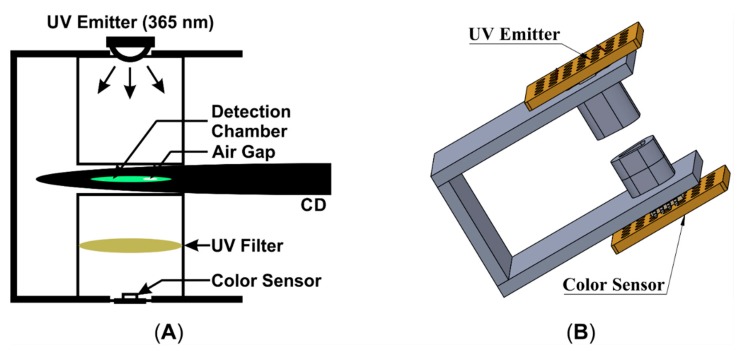
Sensor holder (**A**) Schematic illustration in 2D; (**B**) Schematic illustration in 3D.

The development of the detection system includes: designing of an electronic circuit with suitable electronic components, designing and fabricating a mechanical structure, and developing control program for microcontroller to control and process the whole sequence. The system comprised of several electronic components: a hybrid type stepping motor driven by a motor driver, photointerrupter for accurate positioning the microfluidic CD on target location, a UV emitter (365 nm peak) driven with a LED driver to excite the dye of LAMP amplicons in the detection chamber, a UV filter (optical grade UV glass substrate) to reduce unwanted UV light after excitation of target sample and a color sensor (which includes four groups of photodiode with different color filter, *i.e.*, red, green, blue and clear; Manufacture Part No: TCS3200) to convert the color light intensity to electric pulse train with different frequency levels. A microcontroller (ATmega328) was used for controlling the stepper motor rotation, functioning photointerrupter, processing the color sensor output, and recording the data and displaying the detection result with LCD. The block diagram of the endpoint detection system is shown in Figure 2. Mechanical structure of the system was fabricated with an optical noiseless design using opaque black thermoplastic (*i.e.*, PMMA) (Figure 3). The structure has a sensor holder which houses a UV emitter, a color sensor and a UV filter aligning with the same axis (Figure 4). 

#### 2.3.2. Operation of Detection System

The detection chamber of the microfluidic CD was pre-loaded with the resulting dye of LAMP amplicons and SYBR Green I and then placed on the detection system. On pressing the start button of the system, the CD rotates anti-clockwise until the first chamber is being placed in alignment with the same axis of the UV emitter and color sensor. Then the UV emitter and color sensor are enabled. To process the color sensor output, the microcontroller calculates the mean of 15 continuous pulse durations (due to achieve stable pulse duration reading) from the sensor as single sensor reading for one target. By comparing the sensor reading level with the predefined decision rule for LAMP amplicons detection, a “Positive” or “Negative” text is displayed on the LCD display. Then the motor rotates until the second detection chamber is aligned with the UV emitter and color sensor. Using exactly the same steps, the sensor reads the light intensity level and displays the detection result simultaneously. These steps are being repeated for each of the sixteen detection chambers sequentially. After reading all the samples, the results can be reviewed on the LCD display using navigation keys.

## 3. Results and Discussion

**Figure 5:**
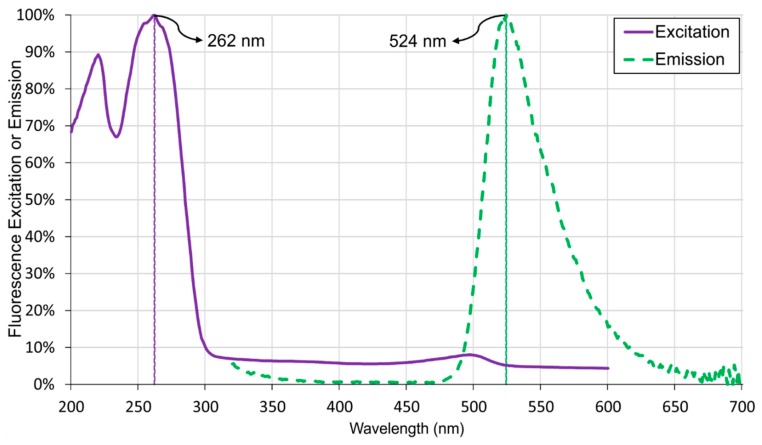
Spectrogram of LAMP Amplicons with *Salmonella* bacteria.

An Epoch Microplate Spectrophotometer and Synergy H1 Hybrid Multi-Mode Microplate Reader were utilized to detect absorbance and fluorescence of the resulting dye of LAMP amplicons with SYBR Green I, respectively. Figure 5 shows the resulting dye absorbs UV light (λ_max_ = 262 nm) and emits green light (λ_max_ = 524 nm). A UV emitter of 365 nm peak wavelength was utilized for excitation of LAMP amplicons rather than 262 nm because of the cost and availability of the component. Moreover, the level of excitation from LAMP amplicons using 365 nm UV emitter is detectable using the color sensor. The group of green photodiodes in color sensor was enabled, because its relative spectral sensitivity is more suitable with the emission spectrum of LAMP amplicons dye as compare to other groups of photodiodes.

**Figure 6:**
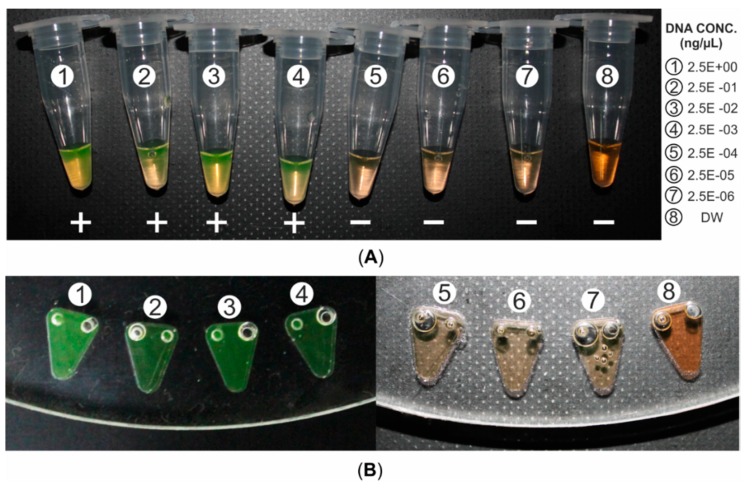
Naked-eye inspection (**A**) Resulting dye (26 µL) of LAMP amplicons (25 µL) and SYBR Green I (1 µL) in ambient light. (**B**) Microfluidic CD loaded with the resulting dye.

**Figure 7:**
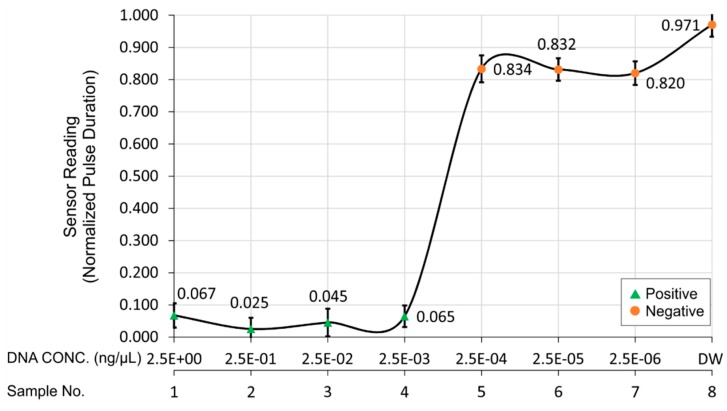
Sensitivity test results of the detection system with different concentrations of *Salmonella* DNA template. The target is classified as positive when the sensor reading is lower than 0.1 and as negative when the sensor reading is higher than 0.8.

A total of seven LAMP reactions were performed with seven different concentrations of DNA templates (10-fold serial dilutions from 2.5 × 10^0^ ng/µL to 2.5 × 10^−6^ ng/µL) of *Salmonella* bacteria and one reaction with deionized water (DW) as a negative control (Figure 6A) and then the dye was transferred in the detection chamber of microfluidic CD (Figure 6B). According to Figure 6A, the initial orange color of SYBR Green I changed to yellowish green in tube number 1 to 4 (positive result), the color turned to faded orange in tube number 5 to 7 (negative result) and the orange color remained unchanged for tube number 8 (negative result). These results indicate the lower limit of detection is 2.5 × 10^−3^ ng/µL for the DNA of *Salmonella* bacteria. As the LAMP detection with SYBR Green I is a qualitative detection process, it gives same intensity of color visually for either positive or negative results. Table 1 and Figure 7 show the sensitivity test results of the detection system when the resulting dye is loaded in the microfluidic CD. The processed sensor output value (pulse duration) as described in the methodology section was normalized with feature scaling method. Table 1 and Figure 7 also illustrate the mean sensor readings with the standard deviation obtained for three different sets of data. These results indicate the normalized value range of sensor output was less than 0.1 (green triangle markers) for the positive control and higher than 0.8 (orange circle markers) for the negative control. As the frequency of sensor output pulse train is proportional to the incident light intensity on the sensor surface and the sensor was operated in green filter mode, which caused higher output pulse frequency and lower output pulse duration for positive control than negative control.

**Table 1:** Sensitivity test results of the endpoint detection system at different concentration of *Salmonella* DNA template.

Sample ID	Concentration of DNA Template (ng/µL) for LAMP Reaction	Color Changes of Resulting Dye **	Visual Identification of Resulting Dye **	Sensor Reading (Normalized Pulse Duration) Mean ± SD	Automatic Detection System Interpretation of Resulting Dye **
1	2.5E+00	Yellowish Green	Positive	0.067 ± 0.038	Positive
2	2.5E-01	Yellowish Green	Positive	0.025 ± 0.035	Positive
3	2.5E-02	Yellowish Green	Positive	0.045 ± 0.043	Positive
4	2.5E-03	Yellowish Green	Positive	0.065 ± 0.033	Positive
5	2.5E-04	Faded Orange	Negative	0.834 ± 0.042	Negative
6	2.5E-05	Faded Orange	Negative	0.832 ± 0.035	Negative
7	2.5E-06	Faded Orange	Negative	0.820 ± 0.037	Negative
8	0 *	Unchanged	Negative	0.971 ± 0.037	Negative

** resulting dye is the dye of LAMP amplicons and SYBR Green I; * DNA template is replaced with DW; SD, standard deviation.

As a parallel assessment, sensitivity test of LAMP assay and PCR assay were tested using 10-fold serial dilutions of pure *Salmonella* cultured DNA. The PCR can detect up to 3.4 × 10^6^ CFU/mL of pure *Salmonella* culture. The detection limit of LAMP on the same set of serially diluted culture was 3.4 × 10^4^ CFU/mL which is 100 times more sensitive than conventional PCR.

Analyzing the sensitivity test results of the detection system with different concentrations of *Salmonella* bacteria DNA, the developed detection system can detect the LAMP amplicons successfully. The current endpoint detection techniques of LAMP amplicons are color changes observation of fluorescent dye with naked eye [52,53,55,56,58] or spectrometer [52,54], accumulated white pellet visualization with turbidimeter after a brief centrifugation of LAMP amplicons [55] and agarose gel electrophoresis showing ladder of DNA bands [58,61]. These methods take 2–5 min or more (depends on the number of LAMP reactions performed) to perform and require presence of personnel to make a confirmative decision (positive or negative). On other hand, this developed detection system can detect 16 samples of LAMP amplicons within 6 s automatically with one hundred percent accuracy. The detection results could be transferred from the detection system to a smart phone and remote places sequentially to improve the contamination monitoring and response time. The developed detection system has succeeded in improving the endpoint detection of LAMP amplicons in terms of time and labor. Moreover, the estimated manufacturing cost of the detection system is less than USD 160 which can be considered as low cost compared to existing detection techniques.

## 4. Conclusions

A color light intensity detection system for LAMP amplicons was designed, implemented and tested for the purpose of automation on a microfluidic CD platform. The developed system could interpret and detect the LAMP amplicons automatically in terms of positive or negative results. The sensitivity test of detection system was performed and detection limit of 2.5 × 10^−3^ ng/µL of *Salmonella* DNA was observed. It could accurately differentiate the positive results from the negative ones. In addition, it could process the sensor reading and display the detection results for 16 samples within 6 s. This detection system enhances the convenience in visualizing the endpoint result as compared to the current naked-eye observation method for LAMP amplicons. This system would be suitable as a point-of-care diagnosis device for rapid detection of foodborne pathogens due to its portability and low manufacturing cost.
